# Albumin adjuvant therapy for acute ischemic stroke with large vessel occlusion (AMASS-LVO): rationale, design, and protocol for a phase 1, open-label, clinical trial

**DOI:** 10.3389/fneur.2024.1455388

**Published:** 2024-09-30

**Authors:** Sihu Pan, Kangjie Du, Shuling Liu, Sifei Wang, Leilei Luo, Yongbo Xu, Chen Cao, Jian Chen, Xunming Ji, Ming Wei

**Affiliations:** ^1^Department of Neurosurgery, Tianjin Huanhu Hospital, Tianjin, China; ^2^Clinical College of Neurology, Neurosurgery, and Neurorehabilitation, Tianjin Medical University, Tianjin, China; ^3^Department of Neurosurgery, Xuanwu Hospital, Beijing, China

**Keywords:** acute ischemic stroke, albumin (ALB), endovascular therapy (EVT), clinical trial, large vessel occlusion (LVO)

## Abstract

**Background:**

Acute ischemic stroke (AIS) is an acute brain injury caused by sudden occlusion of a blood vessel. Endovascular therapy is the most effective way to restore blood flow. However, despite the restoration of blood flow in some patients, their clinical prognosis often remains unsatisfactory. Albumin has shown neuroprotective effects in animal models of AIS. Therefore, this study aims to evaluate the safety, feasibility, and efficacy of local arterial infusions of 20% human serum albumin solution as an adjuvant therapy after endovascular therapy in patients with AIS.

**Methods:**

This study is a prospective, therapeutic exploratory, non-randomized, open-label, phase 1 clinical trial testing the use of 20% human serum albumin solution injected via the artery immediately after successful reperfusion in patients with AIS. The study is divided into two stages. In the first stage, a single-dose-finding will explore the maximum safe dose according to the 3 + 3 dose escalation principle;, with the maximum dose being 0.60 g/kg. After recanalizing the occluded blood vessel, human serum albumin solution will be injected into the internal carotid artery region through a guiding catheter for 30 min. The second stage involves an albumin adjuvant therapy cohort (AT) and an endovascular treatment lonely cohort (ET). The AT cohort will encompass at least 15 additional participants to complete safety trials at the maximum safe dose determined in the first stage. The ET cohort will include well-matched patients receiving endovascular therapy alone, derived from a contemporaneous prospective registry, who will be excluded from having cardiopulmonary disorders and from receiving any neuroprotective therapy. The primary outcome of this study will be symptomatic intracranial hemorrhage. Efficacy outcomes will include the proportion of patients with the progression of cerebral infarction volume, a modified Rankin Scale of 0–2 on day 90 after randomization. An exploratory secondary outcome will be the analysis of thromboinflammatory and neuroprotective molecule profiles.

**Conclusion:**

This pilot trial aims to explore the safety and efficacy of arterial infusion of an albumin solution after occlusive vessel opening in AIS. The results will provide data parameters for subsequent tests on the arterial infusion of albumin solutions.

**Clinical trial registration:**

ClinicalTrials.gov, NCT05953623.

## Background

Having high rates of morbidity, disability, and mortality, stroke is the second leading cause of death worldwide and the main cause of death in China. Ischemic stroke accounts for approximately 70% of all strokes (1, 2) and is a serious threat to human health and well-being. For acute ischemic stroke (AIS), timely and effective restoration of blood flow is one of the main factors influencing good clinical prognosis (3). Endovascular therapy (EVT) can effectively restore occlusive blood flow, significantly reduce the mortality and disability rate of AIS, and is an effective modality to treat AIS (4). However, although >50% of patients who received EVT successfully achieved vascular recanalization, they did not achieve a good clinical prognosis (5, 6). Therefore, adjuvant neuroprotection based on EVT has become a potential treatment for AIS.

The occurrence and development of AIS involve various pathophysiological processes, including excitotoxicity, oxidative stress, inflammatory response, calcium overload, and apoptosis (7, 8). Therapeutic effects can be achieved by intervening in one or more of the pathophysiological processes of AIS. Exploring convenient, safe, and effective neuroprotective therapies is necessary to improve the prognosis of patients with ischemic stroke.

Albumin is a single peptide chain comprising 586 amino acids folded into three domains, with a molecular weight of approximately 67 kDa. It is the most important protein in human plasma, serving various bodily functions such as combining and transporting lipids and lipid-soluble substances, supplementing nutrition, and maintaining plasma colloid osmotic pressure (9). Recently, albumin has been found to exert a neuroprotective effect on the occurrence and development of ischemic stroke. Its neuroprotective mechanisms include antioxidant, anti-inflammation, and anti-edema functions, maintenance of vascular permeability, and regulation of glial cell metabolism (10).

In a mouse model of AIS, a low-dose local arterial infusion of 20% human serum albumin solution was shown to significantly reduce the cerebral infarct volume and alleviate the neurological dysfunction caused by middle cerebral artery occlusion (11). These results suggest that local arterial infusion of 20% human serum albumin solution may benefit the recovery of neurological function after ischemic stroke. However, no clinical trials on local arterial infusion of human serum albumin solution have been conducted to date.

Local intra-arterial administration can help the drug rapidly achieve high local concentrations at the site of action, maximizing its therapeutic effect (12). Based on the neuroprotective effect of albumin and the development of EVT technology, we hypothesize that local arterial infusion of 20% human serum albumin solution will improve nerve function recovery in patients. This study will test the safety, feasibility, and effectiveness of local arterial infusion of 20% human serum albumin solution, based on an intravascular treatment of the responsible occlusive vessels.

## Methods

This study was registered with ClinicalTrials.gov on 20/07/2023 (ID: NCT05953623). The safety of the participants will be monitored continuously by the Ethics Committee of Tianjin Huanhu Hospital.

### Study design

This is a prospective, therapeutic exploratory, non-randomized, open-label, phase 1 clinical trial for testing the use of 20% human serum albumin solution injected via the artery immediately after successful reperfusion in patients with AIS.

### Participants and screening

**Figure 1:**
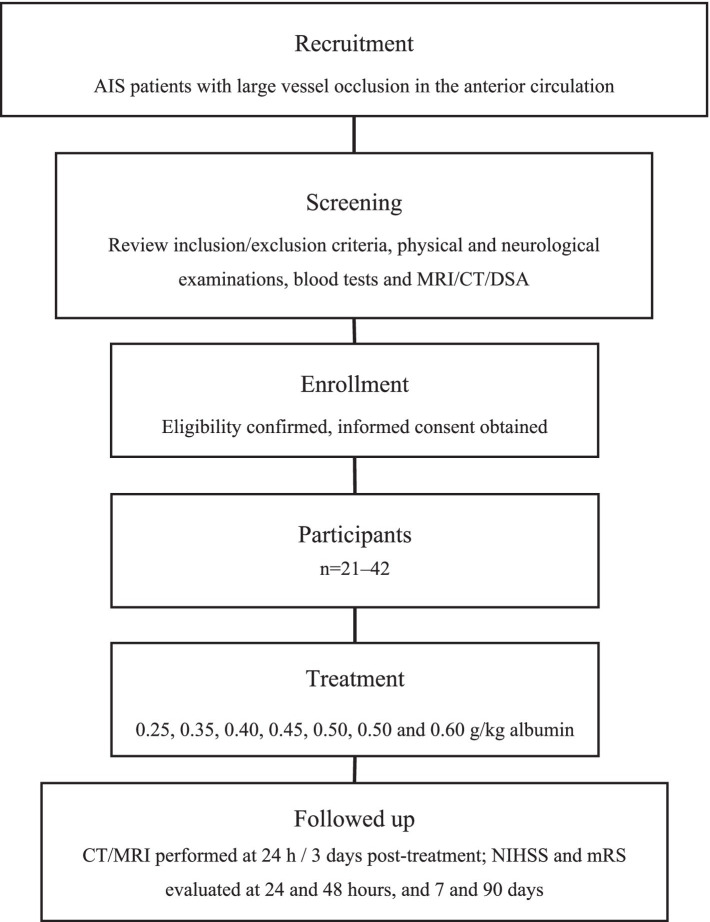
Flowchart of the first stage of the study.

Patients undergoing EVT at the Department of Neurosurgery of Tianjin Huanhu Hospital between August 2023 and May 2024, who meet the enrollment criteria, will be included in this study. Participants will be continuously enrolled into the study group after confirming recanalization of the occluded blood vessels. Each participant or their designated legal representative will review the written informed consent form, which explains the potential procedural benefits and risks. After the participant or the designated legal representative signs the informed consent form, an independent doctor, not involved in the study will perform an evaluation according to the enrollment criteria. If the participant lacks decision-making ability, the designated legal representative will sign the informed consent. The test procedure is shown in Figure 1.

The inclusion criteria are as follows: (1) age 18–80 years; (2) an anterior circulation large vessel occlusion confirmed using computed tomography (CT), magnetic resonance imaging/magnetic resonance angiography (MRI/MRA), or digital subtraction angiography (DSA); (3) a baseline National Institutes of Health Stroke Scale (NIHSS) score ≥6; (4) an arterial puncture was performed within 24 h of stroke onset (defined as either the time the onset of a neurological abnormality was observed the time when symptoms were first noticed on waking from sleep or the last time the patient was observed to be without stroke symptoms); and (5) the occluded vessel reaches an expanded thrombolysis in cerebral infarction (eTICI) score ≥2b after EVT was confirmed using DSA.

The exclusion criteria are as follows: (1) a history of congestive heart failure or jugular dilatation, third heart sound, resting tachycardia due to heart failure (>100 beats/min), or hepatomegaly and lower limb edema without obvious cause on physical examination at admission; (2) hospitalization for acute myocardial infarction within 3 months; (3) symptoms of acute myocardial infarction or admission electrocardiogram data; (4) second- or third-degree heart block or arrhythmia with hemodynamic instability; (5) acute or chronic renal failure (blood creatinine >2.0 mg/dL); (6) severe anemia (hematocrit <32%); (7) clinically suspected aortic dissection; (8) symptoms or CT evidence of subarachnoid hemorrhage; (9) parenchymal cerebral hemorrhage; (10) pregnancy; (11) allergy to albumin; (12) blood pressure >185/110 mmHg on admission; (13) presence of major lung disease; and (14) presence of other life-threatening diseases.

### Randomization and blindness

The study will be divided into two stages: a first-stage dose-escalation test and a second-stage safety test conducted with an expanded sample size based on the first stage. In the first stage, three participants will be included in each dose group according to the 3 + 3 dose increment principle (13). Seven dose groups will be set in this stage: 0.25, 0.35, 0.40, 0.45, 0.50, 0.55, and 0.60 g/kg. As this study is the first to use a local arterial infusion of 20% human serum albumin solution in humans, the highest dose will be set at 0.60 g/kg. Phase 2 will enroll at least 15 additional participants who will be administered the highest safe dose level determined from phase 1 (14). The Data Security Monitoring Board will conduct a mid-term review at the end of phase one. The protocol is designed as an open-label treatment; thus, we will employ partial blinding; that is, the clinical evaluator will be blinded, but the operator and participant will not be blinded.

### Interventions

All participants will be recanalized after completing EVT with responsible occlusive vessels of ≥2b, as confirmed by DSA. Participants will receive 20% human serum albumin solution (China Resources Boya Biopharmaceutical Group Co., Ltd.) corresponding to their dosage group and individual body weight. Albumin will be injected into the diseased side of the internal carotid artery with an infusion time of 30 min. The patient’s blood pressure, heart rate, respiratory rhythm, and oxygen saturation will be monitored throughout the transfusion process. The operative method for the participants will be determined independently by the neuro-interventional physician, who will choose either mechanical thrombectomy or stent insertion according to the intraoperative conditions. All participants will be treated in accordance with the latest Chinese clinical Guidelines for AIS.

### Outcomes and assessment procedures

The participants’ demographic information will be recorded on the case sheet, including previous medical history, time of onset and progression of stroke symptoms, location of responsible occlusive vessels, and course of intravascular therapy. Head MRI and MRA will be performed to determine the location of the cerebral infarction and the occluded blood vessels. All participants will be required to complete chest CT, hematology, and NIHSS testing before enrollment. CT scans of the chest and head will be performed within 2–24 h after EVT. Head MRI will be performed 3 days after surgery and the infarct volume will be calculated and compared to baseline. NIHSS scores will be measured at 24 h, 48 h, day 7, or at discharge. The NIHSS, modified Rankin Scale (mRS), and Barthel index will be assessed at day 90. The participants will undergo hematological examinations at baseline and within 24 h after surgery, including routine blood tests, blood biochemistry, coagulation studies, and cardiac function-related indicators.

Primary outcomes (safety assessment): the primary safety consideration of the trial is the risk of symptomatic intracranial hemorrhage (sICH). The secondary safety outcomes are: (1) intracranial hemorrhage; (2) all-cause mortality; (3) adverse events; (4) serious adverse events; and (5) heart failure, pulmonary interstitial edema, and severe allergic reactions to albumin infusion.

Secondary outcomes (efficacy assessment): (1) the proportion of patients with an mRS of 0–2 on day 90 after surgery; (2) the development of a cerebral infarct on day 3 after surgery; and (3) changes in NIHSS scores on day 7 after surgery.

Exploratory terminal: (1) coagulation function and blood count related to platelet and lymphocyte counts; (2) biochemical markers of thrombophilic inflammation, including interleukin-1α, interleukin-1β, interleukin-6, interleukin-10, interleukin-18, transforming growth factor-β, histamine, 5-hydroxytryptophan, TNF-α, coagulation factor XII (FXII), ADAMTS13, vWF, GP VI, GP IIbIIIa, tissue factor and soluble CD40 ligand (CD40L); (3) plasma proteomic profiling of subjects; (4) detection of antithrombotic properties of different doses of albumin by peripheral blood cultures of PBMC or platelet cultures from patients or volunteers.

### Estimation of sample size

**Figure 2:**
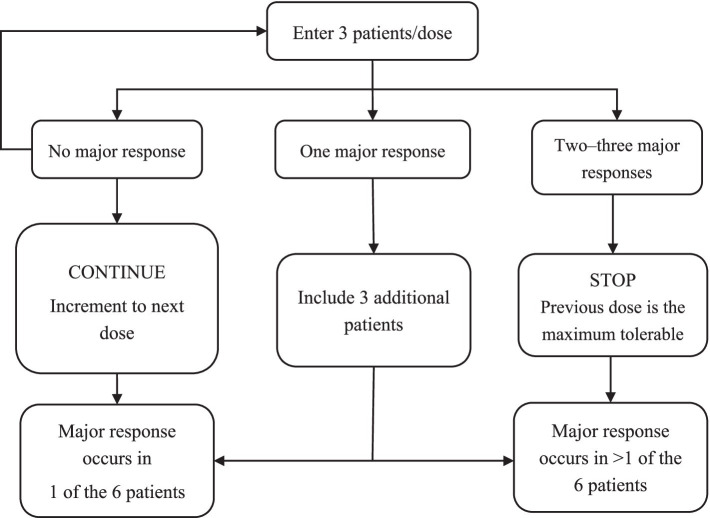
3 + 3 dose-escalation trial design.

This study will adopt the “3 + 3” dose-escalation principle. Seven albumin dose groups will be formed: 0.25, 0.35, 0.40, 0.45, 0.50, 0.55, and 0.60 g/kg. If the primary endpoint is never reached, at least 21 participants need to be included. If one person per dosage group experiences a primary endpoint, 42 participants will be required. A flowchart of the 3 + 3 dose-escalation trial design is shown in Figure 2.

The second phase is a safety trial with an expanded sample size based on the maximum safe dose determined in the first phase. According to a previous study (14), the safety of the albumin solution arterial infusion can be assessed by including 15 participants in the second phase.

### Statistical analyses

Statistical analysis will be conducted based on the intention-to-treat principle. Continuous variables will be described using the mean or interquartile range for normal distribution. Categorical variables will be expressed as the frequency and percentage. In this study, multiple independent groups will be compared. The Kruskal–Wallis *H* test and the Mann–Whitney *U* test will be used for severely skewed continuous variables, and the t-test will be used for normally distributed continuous variables. The chi-squared test or Fisher’s exact test will be used to compare the difference in binary outcomes between the groups. All statistical tests will be conducted using a two-sided test. A value of *p* < 0.05 will be considered statistically significant. All statistical analyses will be performed using SPSS statistical software, version 26.0.

## Discussion

The incidence of acute ischemic stroke is currently increasing yearly worldwide (2). The key to treating AIS is to promptly and effectively protect the ischemic penumbra to preserve the injured brain tissue (15). EVT has become the most effective AIS treatment because it opens the occlusive blood vessels and restores blood flow (16). Although EVT is the most effective vascular recanalization method for patients with AIS complicated with large vessel occlusion, this method is limited by the narrow treatment window, potential surgical complications, and reperfusion injury after vascular recanalization, leading to poor clinical prognoses for patients (17, 18). Developing safe and effective neuroprotection programs is an important research direction in the AIS field to extend the endovascular treatment time window, reduce reperfusion injuries, improve patients’ neurological function, and maximize its therapeutic potential.

The pathophysiology of cerebral ischemia is an extremely complex dynamic process. It involves a series of cascades, including energy failure, excitotoxicity, oxidative stress, and inflammation, which are important in brain injury after cerebral ischemia (7, 19, 20). Neuroprotective therapy can regulate the pathophysiological processes of cerebral ischemia, reduce ischemic penumbra progression, alleviate acute brain injury, and promote functional recovery following AIS (21–23). Numerous neuroprotective regimens have recently been developed, showing good neuroprotective effects in preclinical and clinical trials. For example, atmospheric hyperoxia can cause oxygen molecules to reach the four major regions of cerebral infarction rapidly, thereby reducing the destruction of the blood-brain barrier and improving ischemia-reperfusion microcirculation disorders (24). Low-temperature protection can lower brain cell metabolism, reduce the release of excitatory amino acids, and stabilize the blood-brain barrier and other functions, thereby reducing inflammatory infiltration into cerebral ischemia and alleviating cerebral edema (25, 26). Granulocyte colony-stimulating factor has anti-inflammatory and anti-apoptotic properties, which can contribute to the recovery of nerve function in acute ischemic cerebral infarction (27). Although numerous neuroprotective protocols have been developed and their neuroprotective functions have been demonstrated in animal models and clinical trials, most neuroprotective protocols have not shown similar functions in clinical trials or lack large-scale clinical trials, failing to translate the protocols into clinical treatment (28). Currently, convenient and effective neuroprotective programs are lacking in clinics.

Albumin is widely used in clinics and has been found to have neuroprotective effects in animal models of transient and permanent ischemic stroke (29, 30). Through imaging examination of animal models, the neuroprotective effect of albumin was shown to be related to the normalization of signals on MRI diffusion-weighted imaging and the reduction of necrotic changes in the residual injury area (31). In focal ischemic stroke, albumin can reduce the total infarct volume by two-thirds, reduce cerebral edema by three-quarters, improve microvascular perfusion, transport free fatty acids to the infarct area, and extend the treatment window to 4 h (32–34). An important component of albumin’s protective effect is thought to be exerted through intravascular and anti-inflammatory mechanisms, which include hemodilution; beneficial interactions with the vascular endothelium (8); platelet anti-aggregation (9); antagonism of erythrocyte sedimentation (10) and neutrophil-endothelial binding (11); and binding to the local release of nitric oxide, which reduces the concentration of inflammatory mediators, such as free radicals interleukins.

In preclinical and clinical trials of albumin, intravenous infusion of medium and high doses of 25% human serum albumin solution demonstrates a neuroprotective effect in AIS which increases with increasing doses up to 2.05 g/kg (35). However, including additional participants in phase III showed that intravenous administration of a 25% human serum albumin solution at 2.0 g/kg was not associated with the 90-day clinical outcome but was associated with an increased incidence of cerebral hemorrhage and pulmonary edema (36). In a follow-up study, the negative phase III results were found to be caused by the absence of factors controlling vascular recanalization and the high albumin solution dose. Medium- and high-dose hypertonic human albumin solutions carry the risk of causing serious dose-related adverse reactions. In animal model experiments, local arterial infusion of a low-dose (20%) human serum albumin solution showed a good neuroprotective effect (11).

With the development of EVT, neuroprotective therapies are also changing. Due to high reperfusion success rates, EVT is currently recommended as the basis for any trial evaluating the neuroprotective effect of stroke caused by large vessel occlusion (37). Most recent clinical trials evaluating adjunctive therapies in combination with EVT have shown greater neuroprotective benefits. In clinical trials of local arterial cooling infusion, the experimental group showed a greater improvement in clinical neurological function than the control group (38). Arterial infusion of nitroglycerin significantly improved the clinical prognosis of patients (39).

A 20% solution of human serum albumin is hyperosmolar and can rapidly increase the blood volume by four to five times the amount of infusion (40). A low-dose local arterial infusion of 20% human serum albumin solution can quickly and safely achieve a high drug concentration in the infarction area, producing a greater neuroprotective effect. Before the clinical trial, a 20% human serum albumin solution was injected into the pharyngeal ascending artery area of normal experimental pigs at 0.50 and 0.60 g/kg, in Supplementary Figure S1. The experimental results did not show any cardiopulmonary function impairment. These experimental results will be published with the results of this clinical trial. Following previous results (41) and under the prerequisite of participant safety, the highest dose in this study is set at 0.60 g/kg.

### Limitations

This study is projected to have some limitations. First, the trial is being conducted in a single center with a small sample size, and it does not fully follow the principle of random allocation, possibly leading to selection bias. Second, this study will be the first to perform arterial infusions of an albumin solution; no relevant literature reference could be found. For the safety of participants, the maximum dose of albumin solution in this clinical trial is set at 0.6 g/kg. The dose-effect relationship for arterial infusion of 20% human serum albumin solution remains to be explored.

## Conclusion

This study is a preliminary study designed to explore the safety and efficacy of arterial infusions of 20% human serum albumin solution combined with intravascular therapy in AIS. The main objective of this study is to explore the clinical maximum safe dose and optimal dose for arterial albumin infusion. The results of the study will provide data parameters for further exploration of the efficacy of albumin.
