# Supplementary material for: Albumin adjuvant therapy for acute ischemic stroke with large vessel occlusion (AMASS-LVO): rationale, design, and protocol for a phase 1, open-label, clinical trial
Source: Front Neurol. 2024 Sep 30;15:1455388. doi: 10.3389/fneur.2024.1455388 (PMC11471686; doi:10.3389/fneur.2024.1455388)
Supplement: Supplementary file 1 [file Data_Sheet_1.docx]

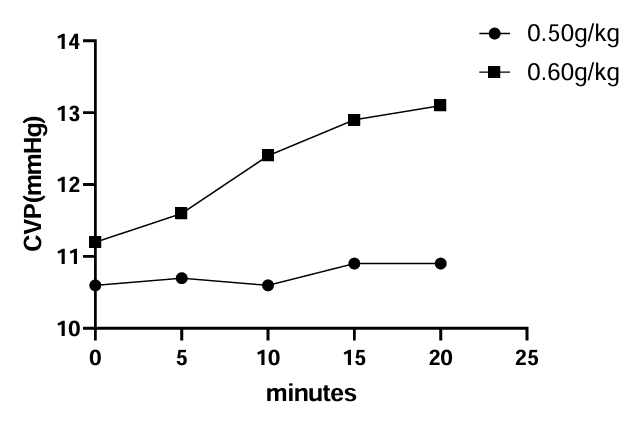


Figure 1 : Curve of central venous pressure and arterial albumin infusion time in experimental animals (Pig). CVP: Central Venous Pressure.
